# Sustained Complete Remission after Radiofrequency Ablation for Liver Oligometastasis from Pancreatic Cancer despite Persistent Carbohydrate Antigen 19-9 Elevation: A Case Report

**DOI:** 10.70352/scrj.cr.26-0003

**Published:** 2026-04-18

**Authors:** Yusuke Takahashi, Akira Kobayashi, Hitoshi Seki

**Affiliations:** Department of Digestive Surgery, Nagano Municipal Hospital, Nagano, Nagano, Japan

**Keywords:** pancreatic ductal adenocarcinoma, liver oligometastasis, radiofrequency ablation, CA19-9, postoperative recurrence, tumor marker kinetics

## Abstract

**INTRODUCTION:**

Persistent elevation of carbohydrate antigen 19-9 (CA19-9) during chemotherapy for pancreatic ductal adenocarcinoma (PDAC) is generally regarded as a marker of poor prognosis and systemic disease, often discouraging aggressive local treatment. However, the clinical significance of elevated CA19-9 levels in patients with oligometastatic recurrence remains controversial.

**CASE PRESENTATION:**

A 69-year-old male underwent subtotal stomach-preserving pancreaticoduodenectomy for resectable pancreatic head cancer, followed by adjuvant S-1 chemotherapy. Thirty-one months after surgery, a solitary 17-mm liver metastasis in segment VI was detected, accompanied by elevated CA19-9 levels. Systemic chemotherapy with gemcitabine plus nab-paclitaxel was administered; however, CA19-9 levels continued to rise despite stable disease on imaging and no evidence of extrahepatic metastasis. Given the persistent CA19-9 elevation suggestive of possible systemic disease, hepatic resection was considered potentially overtreatment. Although radiofrequency ablation (RFA) carries an increased risk of liver abscess in patients with prior biliary reconstruction, it was selected as a less invasive local treatment. The procedure was completed without complications. Following RFA, CA19-9 levels rapidly normalized, and the patient has remained disease-free for more than 3 years without additional chemotherapy.

**CONCLUSIONS:**

This case demonstrates that persistent elevation of CA19-9 levels during chemotherapy does not necessarily preclude long-term disease control through local therapy in selected patients with oligometastatic recurrent PDAC. Treatment decisions should be guided by a comprehensive assessment of tumor biology, metastatic distribution, and clinical context rather than tumor marker levels alone.

## Abbreviations

CA19-9
: carbohydrate antigen 19-9
GnP
: gemcitabine plus nab-paclitaxel
PDAC
: pancreatic ductal adenocarcinoma
RFA
: radiofrequency ablation

## INTRODUCTION

PDAC is an aggressive malignancy with a high rate of postoperative recurrence, even after curative resection.^1)^ Most recurrences represent manifestations of systemic disease; therefore, systemic chemotherapy is recommended as the standard treatment strategy.^2,3)^ In this context, the role of local treatment for postoperative metastases remains controversial.

Serum CA19-9 is widely used as a tumor marker in PDAC and is regarded as a surrogate indicator of tumor burden and treatment response.^4–7)^ Persistently elevated or rising CA19-9 levels during chemotherapy are generally associated with poor prognosis and treatment resistance.^6)^ More recently, markedly elevated CA19-9 levels, such as values exceeding 500 U/mL, have been proposed as indicators of “biological borderline resectable” disease, even in anatomically resectable cases.^8)^ From this perspective, aggressive local treatment is often avoided when tumor marker elevation suggests unfavorable tumor biology.

However, emerging evidence suggests that a subset of patients with limited metastatic burden, referred to as oligometastatic disease, may benefit from local treatment, including surgical resection or ablative therapy.^9,10)^ In particular, isolated liver metastasis after pancreatectomy represents a clinically challenging scenario, in which the optimal balance between oncologic benefit and treatment-related risk remains unclear. This dilemma becomes more complex in patients who have undergone pancreaticoduodenectomy with biliary reconstruction, as local ablative therapies carry an increased risk of infectious complications, such as liver abscess.^11,12)^ In this report, oligometastatic disease refers to 3 or fewer metastatic lesions confined to a single organ without evidence of widespread systemic dissemination.^13)^

Here, we report a rare case of postoperative liver oligometastasis from PDAC in which serum CA19-9 levels continued to rise despite systemic chemotherapy, suggesting unfavorable tumor biology. Nevertheless, RFA of the liver metastasis led to rapid normalization of CA19-9 levels and long-term recurrence-free survival without additional chemotherapy. This case highlights the limitations of tumor marker–based decision-making and suggests a potential role for local therapy even in biologically unfavorable settings.

## CASE PRESENTATION

A 69-year-old male underwent pancreaticoduodenectomy for PDAC located in the pancreatic head. Histopathologic examination confirmed invasive ductal carcinoma with negative surgical margins (R0 resection). Pathological examination revealed a 40-mm primary tumor composed predominantly of well-differentiated adenocarcinoma, with moderately and poorly differentiated components (wel > mod > por); lymphatic, vascular, and perineural invasion were also identified.

**Fig. 1:**
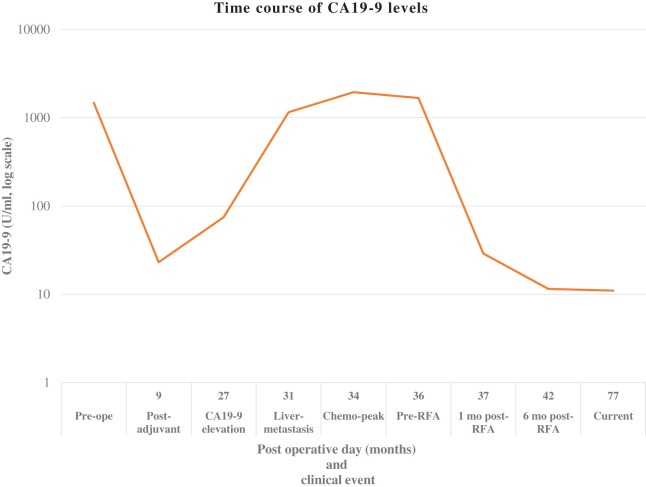
Time course of serum CA19-9 levels. Serum CA19-9 levels markedly decreased after curative resection and adjuvant chemotherapy, followed by a sharp increase at the time of liver metastasis. Despite systemic chemotherapy, CA19-9 levels continued to rise and reached a peak before RFA. Following RFA, CA19-9 levels rapidly normalized and remained stable for more than 3 years without additional chemotherapy. CA19-9, carbohydrate antigen 19-9; RFA, radiofrequency ablation

According to the eighth edition of the Union for International Cancer Control TNM Classification of Malignant Tumors,^14)^ the final pathologic stage was T3N1M0, corresponding to stage IIB. Postoperatively, the patient received standard adjuvant chemotherapy with S-1, after which serum CA19-9 levels decreased from a preoperative value of 1474.7 U/mL to within the normal range (23.1 U/mL; **Fig. 1**).

**Fig. 2:**
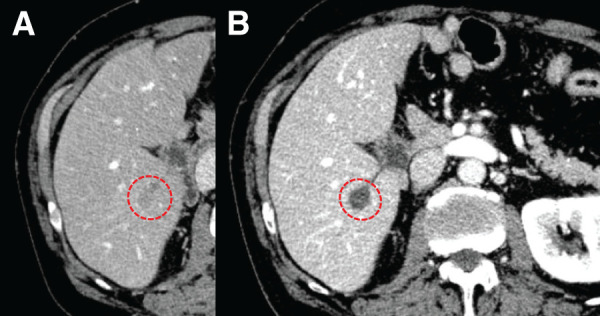
Contrast-enhanced CT images showing liver metastasis from PDAC in segment VI. (**A**) Contrast-enhanced CT image at the time of liver metastasis diagnosis (dotted circle), 31 months after surgery. (**B**) Contrast-enhanced CT image showing the liver metastasis (dotted circle) with no significant change in size after systemic chemotherapy. PDAC, pancreatic ductal adenocarcinoma

At 27 months postoperatively, serum CA19-9 levels showed a first elevation to 74.7 U/mL, and a contrast-enhanced CT performed at 31 months postoperatively revealed a solitary 17-mm liver lesion in segment VI, consistent with metastatic disease (**Fig. 2A**). At the time of diagnosis of liver metastasis, the CA19-9 level was 1153.7 U/mL (**Fig. 1**).

MRI could not be performed because the patient developed claustrophobia during a preoperative MRI examination. No other metastatic lesions were identified. The patient maintained a good performance status. Systemic chemotherapy consisted of GnP administered at 80% dose intensity on a 2-weeks-on/1-week-off schedule for a total of 3 cycles, without dose delay or interruption; however, despite treatment, CA19-9 levels continued to rise, reaching a peak of 1945.8 U/mL (31 months postoperatively) (**Fig. 1**). Radiologic evaluation showed no significant increase in lesion size, and no additional metastatic sites were detected (**Fig. 2B**).

Given the persistent elevation of CA19-9, the possibility of occult systemic disease was considered. Alternative systemic chemotherapy regimens, including modified FOLFIRINOX and 5-FU plus nal-IRI, were discussed with the patient. However, the patient declined any further chemotherapy, expressing a strong preference to discontinue systemic treatment and prioritize QOL. The patient had previously undergone pancreaticoduodenectomy with biliary reconstruction, raising concern about the risk of liver abscess associated with local ablative therapy. Although hepatic resection was discussed as a potential curative option, the invasiveness of surgery in the setting of suspected biologically aggressive disease was considered a significant concern. After a multidisciplinary discussion, RFA was selected as a less invasive local treatment strategy.

**Fig. 3:**
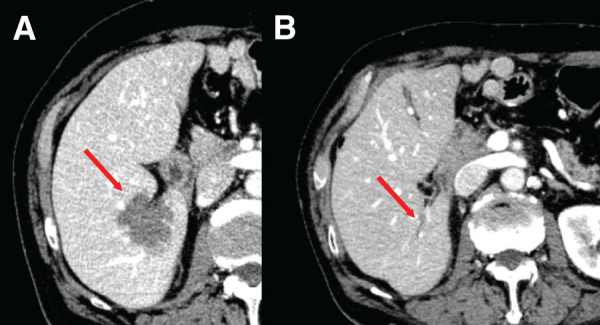
Contrast-enhanced CT images related to RFA. (**A**) Contrast-enhanced CT image obtained immediately after RFA of the liver metastasis. (**B**) Contrast-enhanced CT image showing no evidence of local recurrence more than 3 years after RFA of the liver metastasis. RFA, radiofrequency ablation

RFA was performed without severe complications (**Fig. 3A**). Serum CA19-9 levels were 1673.5 U/mL at 36 months postoperatively before RFA, decreased to 28.9 U/mL 1 month after RFA (37 months postoperatively), and further normalized to 11.5 U/mL at 42 months postoperatively (**Fig. 1**). No adjuvant chemotherapy was administered after RFA. The patient has remained recurrence-free for more than 3 years following RFA, with sustained normalization of CA19-9 levels (11.0 U/mL at 77 months postoperatively) and no evidence of local or distant recurrence on follow-up CT (**Figs. 1** and **3B**).

## DISCUSSION

Postoperative recurrence of PDAC is generally considered a manifestation of systemic disease; therefore, systemic chemotherapy is recommended as the standard treatment strategy.^2,3)^ In particular, elevated serum CA19-9 levels during chemotherapy are commonly interpreted as indicators of treatment failure and poor prognosis.^4–7)^ However, the present case highlights several important clinical issues related to the biological interpretation of tumor marker elevation and the role of local treatment in oligometastatic disease.

In this patient, serum CA19-9 levels normalized after curative pancreaticoduodenectomy and adjuvant chemotherapy but increased markedly at the time of postoperative liver recurrence. Despite the administration of systemic chemotherapy with GnP, CA19-9 levels continued to rise and reached a peak before local treatment, although radiologic evaluation demonstrated only a solitary liver metastasis without apparent progression in size. Such a discrepancy between tumor marker dynamics and imaging findings often raises concern for occult systemic disease, and markedly elevated CA19-9 levels (e.g., exceeding 500 U/mL) have been described as indicative of “biological borderline resectable” disease with unfavorable outcomes.^8)^ From this perspective, aggressive local treatment may be considered futile or even inappropriate.

Nevertheless, accumulating evidence suggests that elevation of CA19-9 does not always reflect widespread systemic dissemination. CA19-9 levels can be influenced by tumor biology, tumor burden, and local tumor activity, and persistent elevation during chemotherapy does not necessarily preclude durable disease control through local therapy.^5,7)^ In the present case, the dramatic and sustained normalization of CA19-9 immediately after RFA strongly suggests that the elevated tumor marker predominantly originated from the liver metastasis itself rather than from occult systemic disease. This observation challenges the assumption that rising CA19-9 levels invariably indicate chemoresistant systemic progression.

In this case, the differential diagnosis of the solitary hepatic lesion included a liver abscess and intrahepatic cholangiocarcinoma. Liver abscess was considered unlikely because the patient had no fever, abdominal pain, or laboratory evidence of inflammation (no elevation of white blood cell counts or C-reactive protein levels) during the period of CA19-9 elevation and lesion detection. Intrahepatic cholangiocarcinoma is histologically an adenocarcinoma, similar to pancreatic cancer, and definitive differentiation between these entities may ultimately require surgical resection with immunohistochemical analysis. However, GnP therapy is generally not effective for intrahepatic cholangiocarcinoma, and the absence of tumor progression after 3 courses of GnP would be inconsistent with its typical clinical course. Taken together, it was reasonable to diagnose the lesion as a metastatic tumor from pancreatic cancer.

The choice of local treatment modality for liver metastasis after pancreaticoduodenectomy requires careful consideration. Hepatic resection has been reported as a potentially curative option in selected patients with isolated liver metastasis from PDAC; however, its indication remains controversial, particularly in patients with high CA19-9 levels suggestive of aggressive tumor biology.^10,13)^ In contrast, the usefulness of RFA for liver metastases from pancreatic cancer has also been reported.^9)^ However, patients who have undergone pancreaticoduodenectomy with biliary reconstruction are at increased risk of liver abscess following local ablative therapies, including RFA, because of potential biliary contamination.^11,12)^ This risk has led some clinicians to favor surgical resection over percutaneous ablation in such settings.

In the present case, persistently rising CA19-9 levels despite chemotherapy raised concern that the disease might not be confined solely to the liver. Performing a highly invasive hepatic resection under these circumstances could have exposed the patient to substantial surgical morbidity without a clear oncologic benefit. After careful multidisciplinary discussion, RFA was selected as a less invasive local treatment that allowed both therapeutic intervention and biological assessment of disease behavior. PET–CT was not performed in this case; however, it might have been a useful option prior to RFA to confirm that the disease was confined to a localized lesion. Although RFA carries a recognized risk of liver abscess formation in patients with biliary reconstruction, this risk was considered acceptable compared with the potential morbidity of major hepatic resection. Importantly, no severe infectious complications occurred, and the patient achieved long-term disease control.

The clinical course of this patient suggests that, even in the presence of markedly elevated CA19-9 levels and apparent chemoresistance, local treatment for postoperative oligometastatic PDAC may be justified in carefully selected cases. The rapid normalization and long-term stability of CA19-9 levels after RFA, together with sustained recurrence-free survival without further chemotherapy, indicate that tumor marker dynamics should be interpreted in conjunction with imaging findings and the clinical context rather than serving as an absolute contraindication to local therapy. Regarding the appropriate size criteria for RFA in hepatic lesions, several clinical trials have defined the indication for thermal ablation as lesions measuring ≤3 cm; therefore, it appears reasonable to consider hepatic tumors ≤3 cm as suitable candidates for RFA.^15,16)^ This case report has inherent limitations due to its single-case nature. Therefore, the findings should be interpreted with caution. Further prospective studies or larger case series are warranted to validate the observations and to better elucidate the clinical implications.

## CONCLUSIONS

This case demonstrates that postoperative liver oligometastasis from PDAC may be effectively controlled with local ablative therapy, even when serum CA19-9 levels continue to rise during systemic chemotherapy. Careful patient selection and multidisciplinary decision-making are essential to optimize outcomes in this challenging clinical scenario. For solitary liver metastases measuring ≤3 cm that do not show progression after systemic chemotherapy, RFA may be considered as a treatment option even in patients who have previously undergone pancreaticoduodenectomy. This case suggests that integrating tumor marker kinetics with imaging-based assessment may provide a more accurate framework for selecting candidates for local therapy in oligometastatic PDAC.

## DECLARATIONS

### Availability of data and materials

All data generated or analyzed during this study are included in this manuscript.
